# Linker Length Matters, Fynomer-Fc Fusion with an Optimized Linker Displaying Picomolar IL-17A Inhibition Potency*

**DOI:** 10.1074/jbc.M113.534578

**Published:** 2014-04-01

**Authors:** Michela Silacci, Nadja Baenziger-Tobler, Wibke Lembke, Wenjuan Zha, Sarah Batey, Julian Bertschinger, Dragan Grabulovski

**Affiliations:** From Covagen AG, Wagistrasse 25, CH-8952 Schlieren, Switzerland

**Keywords:** Drug Discovery, Inflammation, Interleukin, Protein Engineering, SH3 Domains, Fynomer

## Abstract

Fynomers are small binding proteins derived from the human Fyn SH3 domain. Using phage display technology, Fynomers were generated inhibiting the activity of the proinflammatory cytokine interleukin-17A (IL-17A). One specific Fynomer called 2C1 inhibited human IL-17A *in vitro* with an IC_50_ value of 2.2 nm. Interestingly, when 2C1 was genetically fused to the Fc part of a human antibody via four different amino acid linkers to yield bivalent IL-17A binding proteins (each linker differed in length), the 2C1-Fc fusion protein with the longest linker displayed the most potent inhibitory activity. It blocked homodimeric IL-17A with an IC_50_ value of 21 pm, which corresponds to a hundredfold improved IC_50_ value as compared to the value obtained with monovalent Fynomer 2C1. In contrast, the 2C1-Fc fusion with the shortest linker showed only an ∼8-fold improved IC_50_ value of 260 pm. Furthermore, in a mouse model of acute inflammation, we have shown that the most potent 2C1-Fc fusion protein is able to efficiently inhibit IL-17A *in vivo*. With their suitable biophysical properties, Fynomer-Fc fusion proteins represent new drug candidates for the treatment of IL-17A mediated inflammatory conditions such as psoriasis, psoriatic arthritis, or rheumatoid arthritis.

## Teaser

**Background:** IL-17A is a proinflammatory cytokine involved in various inflammatory diseases.

**Results:** Potent IL-17A inhibitors were generated by fusing human Fyn SH3 domain derivatives (Fynomers) to the Fc part of an antibody.

**Conclusion:** The linker length between the Fc part and the binding domain influences significantly the potency of Fc fusion proteins.

**Significance:** Fynomers represent a promising class of therapeutic proteins.

## Introduction

Fc-based fusion proteins represent a major class of therapeutic proteins with seven approved molecules on the market and many others currently under clinical evaluation (1–3). In most cases, therapeutic peptides or proteins are attached to an Fc domain to endow the hybrids with a number of beneficial biological and pharmacological properties (3). Perhaps most importantly, through binding to the neonatal Fc receptor (FcRn), such Fc fusions display IgG-like pharmacokinetic properties (*i.e.* long serum half-life ranging from days to weeks) (4). In contrast, other proteins of ∼70 kDa in size and smaller are typically eliminated rapidly from circulation by renal filtration and have half-lives of a few minutes to a few hours, which can in many cases render them unsuitable for therapeutic applications (5). Beyond half-life extension, Fc fusion can provide several additional benefits such as facilitating expression and secretion of recombinant protein, enabling facile purification by protein A chromatography, binding to Fcγ receptors and/or complement to support secondary immune functions, improving solubility and stability, and enhancing potency by increasing valency (6).

One of the key variables that has to be addressed when engineering an Fc fusion protein is the choice of the linker length and sequence. Many researchers have used a simple glycine and serine (GGGGS)-containing linker as proposed by a study of naturally occurring domain separating linkers (7) or, the naturally ocurring hinge region of an antibody (*i.e.* sequence region between the CH1 and CH2 domains of a full-length antibody), as it is the case for example for the marketed Fc fusion protein etanercept (Enbrel®) (8).

**FIGURE 1.**
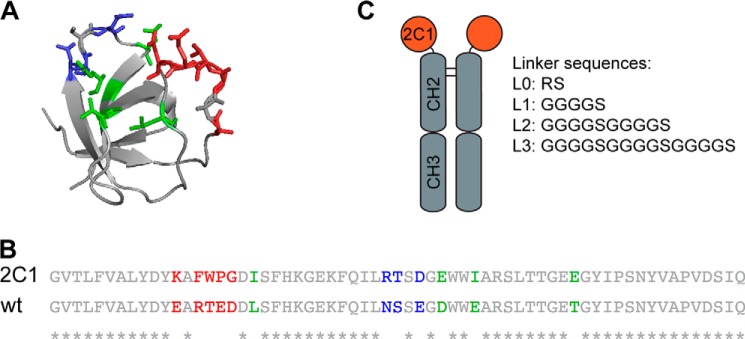
**Fyn SH3 wild-type protein structure, amino acid sequences, and schematic overview on the 2C1-Fc fusion proteins.** *A*, structure of the Fyn SH3 wild type (WT) domain (Protein Data Bank code 1M27) prepared with PyMOL software. Colored amino acid residues in the “stick mode” indicate randomized positions in the Fynomer 2C1. The RT loop is depicted in *red*, the Src loop is shown in *blue*, and mutated amino acid residues in between the loops are colored in *green. B*, alignment of the amino acid sequences of the Fynomer 2C1 and the wild type Fyn SH3 domain (WT). Differences in the RT loop are indicated in *red*, differences in the Src loop are indicated in *blue*, and different amino acid residues in between the loops are marked in *green. C*, schematic picture of the 2C1-Fc fusion protein, showing that the Fynomer 2C1 was genetically fused to the N terminus of the CH2 domain of an antibody-Fc part. Additionally, the amino acid sequences of the four different linkers are shown and named L0–L3, according to the linker length.

In the present article, we show that the linker length plays an important role for the potency of Fc fusion proteins. Using phage display technology (9, 10), we have generated Fynomers inhibiting the activity of the proinflammatory cytokine interleukin 17A (IL-17A). Fynomers are small binding proteins (7 kDa) derived from the human Fyn SH3 domain, which can be engineered to bind to essentially any target of interest with high affinity and specificity (for a review on non-immunoglobulin binding proteins collectively called “scaffolds” (see Refs. 11 and 12). The very stable Fyn SH3 domain (*T_m_* ∼ 70 °C) is a particularly attractive scaffold for the generation of binding proteins because it (*i*) can be expressed in bacteria in soluble form in high amounts, (*ii*) is monomeric and does not aggregate when stored in solution, (*iii*) lacks cysteine residues and, therefore, can be fused to essentially any other protein without misfolding problems, and (*iv*) is of human origin featuring an amino acid sequence completely conserved in different species from mouse to man and was shown to be non-immunogenic in mammals (10). As other SH3 domains, it is composed of two anti-parallel β-sheets and contains two flexible loops (called RT and n-Src loops) to interact with target proteins of interest (Fig. 1*A*). Recently, we have described the generation of large Fyn SH3 phage display libraries and successful isolation of Fynomers binding to the extra domain B of fibronectin (10), albumin (13), chymase (14), BACE-2 (15), and HER2 (16).

In the present study, we used IL-17A as target because it is a proinflammatory cytokine that has been implied in many autoimmune diseases such as rheumatoid arthritis (17), psoriasis (18), Crohn's disease (19), multiple sclerosis (20), and lupus erythematosus (21). Several anti-IL-17A inhibitors have been shown *in vitro* and *in vivo* to reduce the release of innate immune effectors and are currently being investigated in clinical trials for the treatment of several inflammatory conditions such as rheumatoid arthritis, uveitis, and psoriasis (22–24).

Here, we describe the Fynomer 2C1, which inhibits human IL-17A *in vitro* with an IC_50_ value of 2.2 nm. Interestingly, when 2C1 was genetically fused to the Fc part of a human antibody via four different amino acid linkers to yield bivalent binding proteins (each linker differed in length, see Fig. 1*C*), the 2C1-Fc fusion protein with the longest linker displayed the most potent inhibitory activity, blocking homodimeric IL-17A with an IC_50_ value of 21 pm, which corresponds to a hundredfold improved IC_50_ value as compared to the value obtained with monovalent Fynomer 2C1. In contrast, the 2C1-Fc fusion with the shortest linker showed only an ∼8-fold improved IC_50_ value of 260 pm, which can be explained due to increased valency of the Fc fusion. Furthermore, in a mouse model of acute inflammation, we have shown that the most potent 2C1-Fc fusion protein is able to efficiently inhibit IL-17A *in vivo*. With their suitable biophysical properties, Fynomer-Fc fusion proteins represent new drug candidates for the treatment of IL-17A mediated inflammatory conditions such as psoriasis, psoriatic arthritis, or rheumatoid arthritis.

## EXPERIMENTAL PROCEDURES

### 

#### 

##### Isolation of Fynomers using Phage Display Libraries

Using a Fynomer phage display library (10), Fyn SH3-derived binding proteins (Fynomers) specific to IL-17A were isolated using recombinant IL-17A (R&D Systems) as antigen and standard phage display as selection technology (9, 10). After selections, monoclonal bacterial supernatants containing phage were used for phage ELISA (9). Fynomers specifically binding to IL-17A were used as templates for cloning affinity maturation libraries. The process of affinity maturation library generation was essentially the same as described by Schlatter *et al.* (14) for cloning of the naïve library with randomizations in the RT loop, Src loop, or outside of the loops. After affinity maturation selections, Fynomers were screened for binding to IL-17A by lysate ELISA. Briefly, DNA encoding the Fyn SH3-derived binding proteins were cloned into the bacterial expression vector pQE12 (Qiagen) resulting in C-terminal Myc-His_6_-tagged constructs as described previously (10). The polypeptides were expressed in the cytosol of *Escherichia coli* bacteria in a 96-well format, and 200 μl of cleared lysate was used for ELISA as described previously (13). The DNA sequence of the specific binders was verified by DNA sequencing (Microsynth).

##### Fynomer 2C1 Expression and Purification

Monomeric Fynomer 2C1 (Fig. 1*B*) was subcloned into the expression vector pQE12 and therewith fused to a Myc and His_6_ tag (14). After expression, 2C1 was purified using nickel affinity chromatography (Qiagen) according to the manufacturer's instructions. Protein size and purity was assessed by SDS-PAGE analysis under non-reducing conditions. 15 μl of a 0.4 mg/ml protein solution was mixed with 5 μl of loading buffer and 15 μl mixture was loaded (NuPAGE® Novex 4–12% Bis-Tris gel, Invitrogen). To determine the protein size, 5-μl full-range rainbow molecular weight marker was used (GE Healthcare). Proteins were allowed to segregate in NuPAGE® MES SDS running buffer (Invitrogen), stained with Coomassie staining solution and destained in deionized water.

Protein aggregation state was analyzed using a ÄKTA purifier system (GE Healthcare). 100 μl of a 0.34 mg/ml protein solution was loaded and analyzed on a Superdex 75 10/300 GL column (GE Healthcare).

##### Affinity Measurements

Affinity measurements were performed using a BIAcore T200 instrument (GE Healthcare). For the interaction analysis between 2C1 monomer and IL-17A, a Series S CM5 chip (GE Healthcare) was coated with 2000 Resonance Units of IL-17A immobilized using the amine coupling kit (GE Healthcare). The running buffer was PBS containing 0.05% Tween 20. The interactions were measured at a flow of 30 μl/min and injection of different concentrations of Fynomer 2C1 (highest concentration, 100 nm and subsequent 3-fold dilution series). All kinetic data of the interaction were evaluated using the BIAcore T200 evaluation software. Similarly, for the interaction analysis between 2C1-Fc fusions and IL-17A, a Series S CM5 chip was coated with 500 Resonance Units of IL-17A immobilized using the amine coupling kit (GE Healthcare). The interactions were measured at a flow of 30 μl/min (PBS containing 0.05% Tween 20) and injection of different concentrations of 2C1-Fc fusions (highest concentration: 50 nm and subsequent 2-fold dilution series). All kinetic data of the interaction were evaluated using the BIAcore T200 evaluation software.

##### Specificity ELISA

**FIGURE 2.**
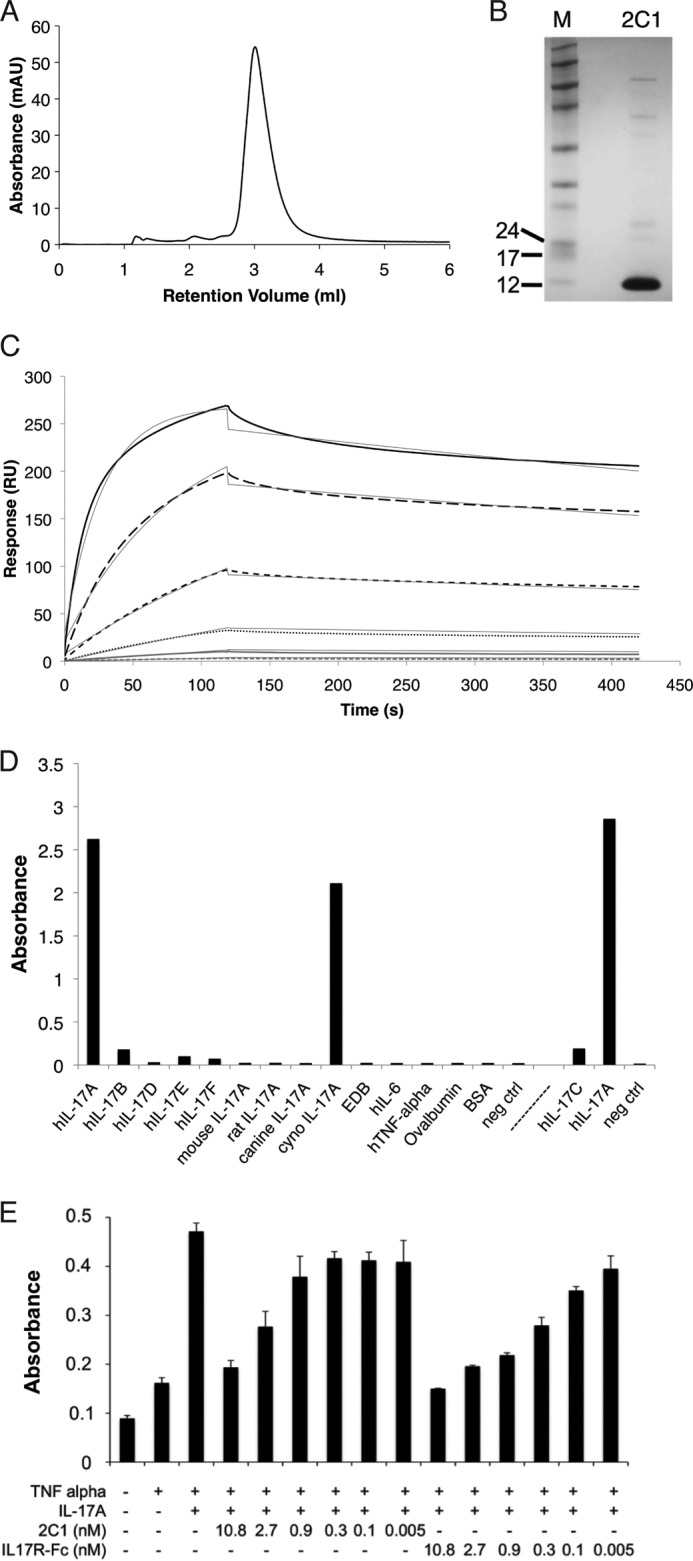
**The Fynomer 2C1 exhibits excellent biophysical properties and a high affinity and specificity to its target protein IL-17A.** *A*, the monomeric Fynomer was expressed in *E. coli* and purified via a His_6_ tag affinity chromatography. The resulting protein was 95% pure and monomeric (*M*), as determined by SEC and SDS-PAGE (*B*). *C*, real-time interaction analysis on a BIAcore chip coated with IL-17A was used to determine the dissociation constant (*K_D_*). Different concentrations of Fynomer 2C1 (highest concentration, 100 nm and subsequent 3-fold dilution series) were injected revealing a *K_D_* value of 1.8 nm at the antigen surface density used. *D*, to investigate binding specificity of the monomeric Fynomer 2C1, indicated proteins were coated onto an ELISA plate. 2C1 bound human and cynomolgus (*cyno*) IL-17A in a specific manner and did not bind to any of the other IL-17 family members or IL-17A cytokines from other species nor to unrelated proteins. Binding to IL17-C was determined in a separate experiment, indicated by the *dashed line. neg ctrl*, negative control; *EDB*, extradomain B of fibronectin. *E*, 2C1 specifically inhibited IL-17A in a dose-dependent manner. As a positive control for IL-17A inhibition, commercially available IL-17R-Fc fusion was used.

The target proteins human IL-17A (R&D Systems), human IL-17B (Peprotech), human IL-17C (R&D Systems), human IL-17D (Peprotech), human IL-17E (Peprotech), human IL-17F (AbD Serotec), mouse IL-17A (R&D Systems), rat IL-17A (Akron Biotech), canine IL-17A (R&D Systems), human IL-6 (R&D Systems), human TNF-α (Piercenet), ovalbumin (Sigma), bovine serum albumin (BSA, Sigma) cynomolgus IL-17A (produced in-house), extra domain-B of fibronectin (25) were coated onto MaxiSorp® plates (Nunc) at a concentration of 10 μg/ml. After blocking of residual binding sites with 2% milk in PBS (v/w, Rapilait, Migros, Switzerland), 2C1 monomer was incubated at 80 nm with the antibody 9E10 (Roche Applied Science, 3 μg/ml) in 2% milk in PBS. After washing, the wells were incubated with goat anti-mouse IgG in 2% milk in PBS (1:1000, Sigma, HRP-conjugated). After washing, BM Blue POD (Roche Applied Science) was added as substrate for color development. This reaction was stopped after 5 min with 1 m sulfuric acid (Sigma). Binding to human IL-17C was determined in a separate experiment, indicated by the *dashed line* (Fig. 2*D*). For each condition, the reference-subtracted absorbance was calculated by *A*_450_ − *A*_650_ (reference wavelength) using a SpectraMax® M5e Microplate Reader (Molecular Devices). The data were imported into Excel (Microsoft), and the average of the duplicates was calculated.

##### Cloning, Expression, Purification, and Quality Control of 2C1-Fc Fusions

The four different 2C1-Fc fusion proteins were cloned and expressed by the CRO Evitria AG (Schlieren, Switzerland). For purification, 250 ml of cell culture supernatant was applied onto a Mabselect SuRe column (GE Healthcare) using an ÄKTA purifier system (GE Healthcare). The column was washed with 15 column volumes of PBS, pH 7.4, and the protein was eluted using 0.1 m glycine, pH 2.8, collecting 1-ml fractions. After elution, pH was adjusted with 1 m Tris, pH 9. The OD of the fractions was determined using an Infinite M200 pro reader and a NanoQuant plate^TM^ (Tecan). The two fractions showing the highest OD were loaded onto an ÄKTA purifier. Preparative size exclusion was performed using a Superdex 200 10/300 GL column (GE Healthcare), and the storage buffer was exchanged to phosphate-buffered saline (PBS, pH 7.4, Invitrogen). The four highest OD fractions were combined and used for further studies. Protein size, purity, and aggregation state was analyzed using an ÄKTA purifier system (GE Healthcare).

##### ELISA Competition Experiments

All four 2C1-Fc fusion proteins were tested in ELISA competition experiments to determine their capabilities to block the interaction between IL-17A and its receptor. Maxisorp plates (Nunc) were coated with commercially available IL-17R-Fc fusion (R&D Systems) overnight at a concentration of 0.6 μg/ml. After blocking with 1% BSA in PBS, different concentrations of the Fc fusions were pre-incubated with 2.5 nm of biotinylated IL-17A (R&D Systems; biotinylated in-house with NHS-PEG4-biotin according to the manufacturer's instructions (Pierce)); and added to the wells. After washing, the wells were incubated with streptavidin-HRP conjugate (R&D Systems) 1:200 diluted in 1% BSA/PBS. After washing, BM Blue POD (Roche Applied Science) was added as substrate for color development. This reaction was stopped after 5 min with 1 m sulfuric acid (Sigma). For each condition, the reference-subtracted absorbance was calculated by *A*_450_ − *A*_650_ (reference wavelength) using a SpectraMax® M5e Microplate Reader (Molecular Devices). IC_50_ values were calculated using Prism software (version 5).

##### IL-17A Inhibition Cell Assays

The inhibitory potencies of monomeric 2C1 and all four 2C1-Fc fusion proteins were determined by stimulating normal human dermal fibroblasts with recombinant IL-17A and TNF, as they act in synergy (26). Normal human dermal fibroblast cells (ATCC PCS-201-010) were seeded in 96-well plates (TPP) in fibroblast growth medium (Promocell). The cells were allowed to adhere. Supernatant was exchanged with medium containing IL-17A (34 pm, R&D Systems) and TNF (1.2 pm, Pierce), with and without the anti-IL-17A compounds (2C1, 2C1-Fc fusions, and commercially available IL-17R-Fc (R&D Systems)). As negative control, equal volume of PBS was added to the medium. Additionally, cells were treated with TNF only to mimic IL-6 levels after full IL-17A inhibition (“TNF control”). After 24 h, supernatants were collected, filtered (0.45 μm pore size), and used to determine IL-6 concentrations by ELISA according to the manufacturer's instructions (DuoSet, R&D Systems). Data points were measured in triplicate. Data indicate a representative result. The percentages of inhibition were plotted, and IC_50_ values were calculated using Prism software (version 5). The percentage of IL-17A inhibition was determined with the following formula.

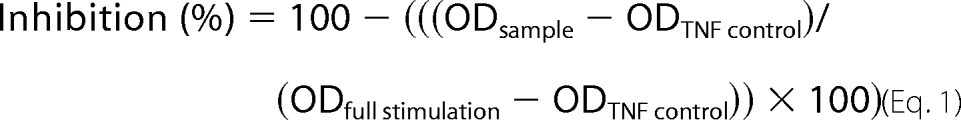


##### Mouse Pharmacokinetic Study

2C1L_3_Fc solution was injected i.v. into five mice (C57BL/6, Charles River) at a dose of 1.5 mg/kg. After the indicated time points, ∼20 μl of blood were taken from the vena saphena with the capillary Microvette CB 300 (Sarstedt). The blood samples were centrifuged for 10 min at 9500 × *g*, and the serum was stored at −20 °C until ELISA analysis was performed. Serum concentration was determined by ELISA using a reference standard of 2C1L_3_Fc. 50 μl biotinylated IL-17A (30 nm) (R&D Systems, biotinylated using NHS-PEO4-biotin (Pierce) according to the manufacturer's instructions) was added to streptavidin-coated wells (Reactibind, Pierce) and after blocking with PBS, 4% milk (Rapilait, Migros, Switzerland), 45 μl of PBS, 4% milk, and 5 μl of serum sample were added. After incubation for 1 h and washing, bound Fc fusion proteins were detected with protein A-HRP conjugate (Sigma). Peroxidase activity was detected by addition of QuantaRed enhanced chemifluorescent HRP substrate (Pierce). Fluorescence intensity was measured after 5 to 10 min at 544 nm (excitation) and 590 nm (emission). From the concentrations determined in serum at different time points and the resulting slope *k* of the elimination phase (plotted in a semi-logarithmic scale), the half-life of 2C1L_3_Fc was calculated using to the formula *t*_½_ = ln2/−*k*.

##### Mouse Efficacy Study

Human IL-17A binds and stimulates the murine receptor resulting in an increase of serum KC-chemokine (CXCL1) levels (31). To test the most potent molecule 2C1L_3_Fc *in vivo*, C57BL/6NCrl mice (Charles River) were injected intravenously with 2C1L_3_Fc at a dose of 0.5 mg/kg or PBS only. One hour post administration, 3 μg/mouse of human IL-17A or PBS was injected subcutaneously. At 2 h after treatment, blood was collected (Microvette CB 300, Sarstedt), and sera were stored frozen until analysis. Five mice were used per treatment group.

KC levels were determined using the Quantikine® mouse CXCL1/KC immunoassay (R&D Systems) according to the manufacturer's instructions. Briefly, 25 μl of sera were mixed with 25 μl of calibrator diluent as described. Then, 50 μl of sample diluent were added. Statistical analyses were performed using GraphPad Prism software (version 6, GraphPad Software) and unpaired *t* test assuming Gaussian distribution. A *p* value < 0.05 was considered as statistically significant. All animal studies were approved by the Veterinaeramt des Kantons Zurich (Zurich, Switzerland, license no. 54/2008).

## RESULTS

### 

#### 

##### Isolation and Characterization of Fynomer 2C1 Inhibiting IL-17A

Fynomers specific to human IL-17A were isolated by standard phage display selections (10). After few rounds of panning on biotinylated IL-17A as target, several Fynomers were identified by phage ELISA. These Fynomers were used as templates for further affinity maturation strategies, introducing new amino acid randomizations in either the RT or Src loop and/or selected amino acids near the loop regions, resulting in the isolation of Fynomer 2C1 (Fig. 1*B*). Fynomer 2C1 was subcloned into a cytosolic expression vector as a monomer fused to a His_6_ and Myc tag, expressed and purified using nickel affinity chromatography. 2C1 could be expressed with a high yield of 70 mg/liter under non-optimized conditions in shake flasks, and the resulting protein was 95% pure and monomeric, as determined by size exclusion chromatography (SEC)^4^ and SDS-PAGE (Fig. 2, *A* and *B*). The affinity of 2C1 toward its target antigen was analyzed by real-time interaction analysis on a BIAcore chip coated with IL-17A, revealing a dissociation constant (*K_D_*) of 1.8 nm at the antigen surface density used (Fig. 2*C*). 2C1 bound human and cynomolgus IL-17A in a specific manner and did not cross-react with any of the other IL-17 family members or IL-17A cytokines from other species, and it did not bind to unrelated proteins as determined by ELISA (Fig. 2*D*). The cell-based inhibition assays identified Fynomer 2C1 as a potent IL-17A inhibitor with an IC_50_ value of 2.2 nm (Fig. 2*E*). Notably, the cell based inhibition assay used both IL-17A and TNF to stimulate the release of IL-6 by normal human dermal fibroblasts as described under “Experimental Procedures.” As expected, 2C1 was able to inhibit specifically IL-17A-induced IL-6 release. As a control, commercially available IL-17-receptor (IL-17R)-Fc fusion was used inhibiting IL-17A activity with an IC_50_ value of 0.6 nm (Fig. 2*E*).

##### Generation of Different 2C1-Fc Fusion Proteins Showing Excellent Biophysical Properties

**FIGURE 3.**
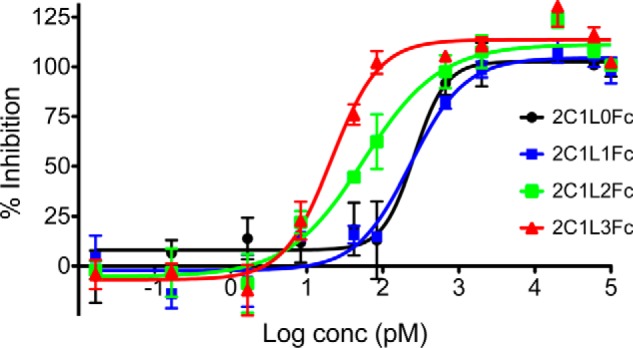
**2C1-Fc fusion molecules show linker-length dependent IL-17A inhibition *in vitro*.** 2C1 was genetically fused to the Fc-part of a human IgG1 using four linkers of variable length (L0–L3, see Fig. 1*C*). The Fc-fusion molecules were tested in a cell assay after stimulation with IL-17A and TNF. The Fynomer-Fc fusion proteins with longer and more flexible linkers, 2C1_L2_Fc and 2C1_L3_Fc, showed most potent inhibition of IL-17A. *log conc*, log concentration.

Fynomer 2C1 was genetically fused to the Fc part of a human antibody because of two reasons. First of all, 2C1 has a molecular mass of ∼7 kDa, which is below the renal clearance threshold of 65 kDa, and therefore, it would be rapidly cleared through the kidneys upon injection into animals or humans. The preparation of Fc fusion proteins of otherwise short-lived molecules represents an attractive avenue to prolong the *in vivo* half-life (6, 27). Second, because IL-17A is a homodimeric protein, we wanted to investigate whether not only valency could be increased but also avidity could be introduced into the binding interaction between 2C1 and IL-17A, *i.e.* two 2C1 Fynomers binding to one homodimeric IL-17A molecule. It was speculated that increased avidity would result in an increased neutralizing potential of 2C1-Fc fusion proteins. But *a priori*, it was not clear which linker length between the Fynomer and the Fc part would allow for avid binding to IL-17A. Therefore, four different linkers with variable length were chosen (linker L0, RS; L1, (GGGS)_1_; L2, (GGGS)_2_; L3, (GGGS)_3_; Fig. 1*C*). The molecules were expressed in a transient CHO expression system and purified using a two-step method: protein A column followed by preparative SEC. All constructs could be expressed with approximately the same yield (L0, 12.2 mg/liter; L1, 10.3 mg/liter; L2, 22.4 mg/liter; L3, 20.7 mg/liter). The purity, size, and aggregation state were analyzed by SEC, indicating that all purified proteins were >95% pure and monomeric. Affinity measurements and ELISA competition assays with the four Fc fusion proteins showed that the 2C1-Fc fusion with the longest linker had the highest affinity to IL-17A and could block the interaction between IL-17A and its receptor most efficiently (Table 1). Importantly, all four Fc fusion molecules were tested in the bioactivity cell assay as described above, as the conditions of this assay allow to detect low IC_50_ values because IL-17A is used at a very low concentration (34 pm). As expected, all 2C1-Fc constructs showed an increased potency as compared with the monomeric Fynomer 2C1 because of the increased valency (21–260 pm *versus* 2.2 nm, respectively, Figs. 3 and 2*E*). Interestingly, only the longer and more flexible linkers of 2C1_L2_Fc and 2C1_L3_Fc improved the IC_50_ values by approximately a factor of forty to one hundred (56 pm and 21 pm, respectively) as compared with the monovalent 2C1 Fynomer, indicating that avidity effects contribute to the increased potency. The Fc fusion proteins with the shorter linkers (2C1_L0_Fc and 2C1_L1_Fc) could only increase the potency by a factor of about eight to nine (260 pm and 232 pm, respectively, Fig. 3).

**TABLE 1:** **Dissociation constants and IC_50_ values of the four different 2C1-Fc fusion proteins** The affinity of the Fc fusion proteins to IL-17A was determined by Biacore. In ELISA competition experiments, the IL-17 receptor was immobilized on a plate and different concentrations of the Fc fusions were pre-incubated with biotinylated IL-17A (2.5 nm) and added to the wells. Bound IL-17A was detected with streptavidin conjugate. 2C1_L3_Fc blocked the interaction between IL-17R and IL-17A most efficiently.

Fusion construct	Affinity (*K_D_*)	ELISA competition (IC_50_)
	*pm*	*pm*
2C1_L0_Fc	450	1809
2C1_L1_Fc	357	1125
2C1_L2_Fc	309	842
2C1_L3_Fc	276	525

##### In Vivo Experiments with 2C1_L3_Fc, Pharmacokinetics and IL-17A Inhibition

**FIGURE 4.**
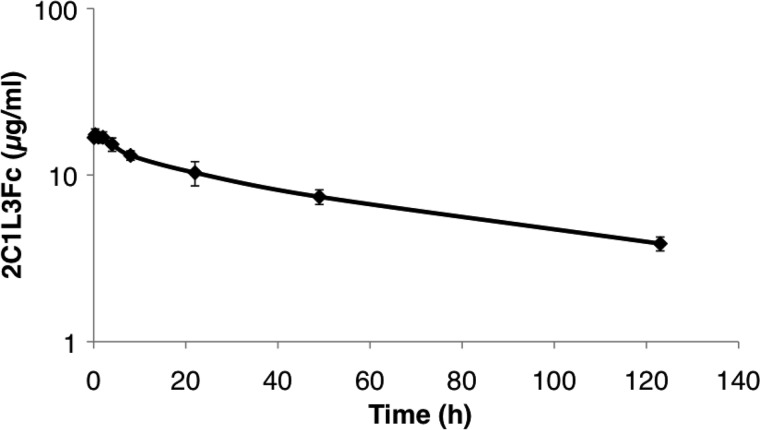
**Serum concentrations of 2C1_L3_Fc in mice.** Serum concentrations in serum are plotted in a semi-logarithmic scale *versus* time after a single intravenous injection in mice.

**FIGURE 5.**
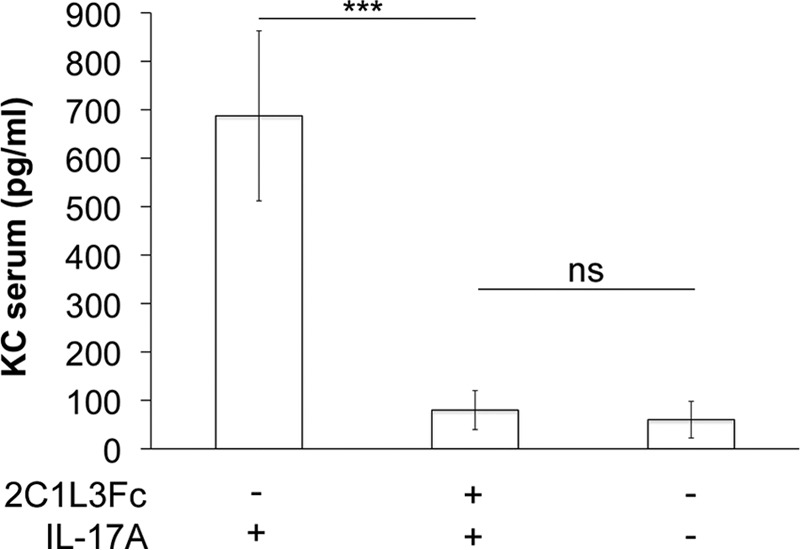
**IL-17A inhibition *in vivo* by 2C1_L3_Fc.** Intravenously injected 2C1_L3_Fc completely inhibited IL-17A-induced KC up-regulation in mice. Mean KC levels of five mice per group are shown (± S.D.). ***, *p* < 0.0001. *ns*, not significant.

The *in vivo* half-life of 2C1_L3_Fc was determined by measuring serum concentrations at different time points after a single i.v. injection in mice by ELISA. A half-life value of 68 h was calculated from the elimination phase (Fig. 4). Mice treated with human IL-17A upregulate the chemokine KC, which can be measured in serum (31). We here investigated whether 2C1L_3_Fc can inhibit IL-17A-mediated KC up-regulation *in vivo*. In the experiment, intravenously injected 2C1L_3_Fc completely abrogated KC induction by subcutaneously administered human IL-17A (*p* < 0.0001, Fig. 5), demonstrating the ability of 2C1L_3_Fc to inhibit human IL-17A in an *in vivo* setting.

## DISCUSSION

IL-17A has been identified as a cytokine that contributes to inflammatory symptoms in autoimmune diseases. IL-17A is found at elevated levels in synovial fluid of patients with rheumatoid arthritis and has been shown to be involved in early rheumatoid arthritis development. IL-17A is a potent inducer of TNF-α and IL-1, the latter being mainly responsible for bone erosion, which has very painful consequences for affected patients (28). Furthermore, inappropriate or excessive production of IL-17A is associated with the pathology of various other diseases and disorders, and therefore, much effort has been invested by the pharmaceutical industry to identify molecules capable of inhibiting the detrimental effects of IL-17A, some of which are currently being tested in clinical trials for different inflammatory conditions (22, 23) such as Crohn's disease, psoriatic arthritis, ankylosing spondylitis, multiple sclerosis, and polymyalgia rheumatica (www.clinicaltrials.gov).

This study describes a Fynomer derived from the Fyn SH3 domain (10) that inhibits IL-17A *in vitro* and *in vivo* with a very high potency. The IL-17A inhibitory Fynomer was isolated from a phage display library with subsequent affinity maturation. The soluble Fynomer has good biophysical properties as determined by SDS-PAGE, SEC, and BIAcore. Using a normal human dermal fibroblast cell IL-17A inhibition assay, the potency of 2C1 was measured to be 2.2 nm, which is a satisfactory value, considering the monomeric nature of the compound and in comparison with the bivalent IL-17RA-Fc fusion that inhibited IL-17A with a potency of 0.6 nm.

The fusion of an Fc portion to 2C1 was expected to have the dual benefits of both an increase in pharmacokinetic through FcRn binding and an increase in the observed affinity and potency of IL-17A inhibition through avid binding to the homodimeric ligand. The observed β-half-life of 68 h *in vivo* corresponds well to previously reported data of human Fc fusion proteins in mice (29). The expected increase in potency due to avid binding of a bivalent inhibitor to a dimeric or multimeric ligand has been reported to be in the range of up to 500-fold (30). Because *a priori*, the optimal linker length and degree of flexibility needed between 2C1 and the Fc part to allow for highly avid binding to IL-17A were not known, we constructed a series of four Fc fusion proteins comprising linkers of variable length, thereby increasing stepwise the flexibility in the hinge region of the anti-IL-17A Fc fusion proteins. Affinity measurements and ELISA competition assays demonstrated the superiority of the 2C1-Fc fusion with the longer linker, as they had higher affinities to IL-17A and could block the interaction between IL-17A and its receptor more efficiently (Table 1). The results of the inhibition cell assay further showed that the introduction of short linkers (either the amino acids RS or GGGGS) was not sufficient to allow for a highly avid binding mode as the potency did not increase significantly (IC_50_ values of 2.2 nm, 260 pm, or 232 pm for monomeric 2C1 or 2C1-Fc fusions with RS or GGGGS linkers, respectively). Increased flexibility due to longer linkers of 10 or 15 amino acids, (GGGGS)_2_ and (GGGGS)_3_, led finally to an up to 105-fold increase in activity, resulting in a highly potent IL-17A inhibitor (2C1_L3_Fc) with an IC_50_ value of 21 pm. Importantly, in a murine acute inflammation model 2C1_L3_Fc was shown to inhibit IL-17A *in vivo*. These data exemplify an important aspect of Fc fusion biotechnology, *e.g.* the ability to engineer a drug candidate for needed potency and valency. In addition, we expect 2C1_L3_Fc to be a selective inhibitor of the IL-17A/A homodimer with a preference over the IL-17A/F heterodimer, due to the avid binding mode toward the IL-17A/A homodimer.

In summary, we have described the isolation of an anti-IL-17A Fynomer, and by using the inherent flexibility of the technology platform, we have been able to use Fc fusion formatting to produce one of the most potent IL-17A inhibitors described to date. A systematic study of increased linker length between the binding Fynomer and the Fc region showed that a critical length of linker is required to provide full flexibility to allow simultaneous binding to both chains of the IL-17A homodimer. The encouraging results described herein are highly promising for further preclinical and clinical development of 2C1_L3_Fc for the treatment of different inflammatory conditions.

## Footnotes

4 The abbreviation used is:

SEC: size exclusion chromatography.
